# An Update of Kaempferol Protection against Brain Damage Induced by Ischemia-Reperfusion and by 3-Nitropropionic Acid

**DOI:** 10.3390/molecules29040776

**Published:** 2024-02-08

**Authors:** Carmen López-Sánchez, Ricardo Lagoa, Joana Poejo, Virginio García-López, Virginio García-Martínez, Carlos Gutierrez-Merino

**Affiliations:** 1 Institute of Molecular Pathology Biomarkers, University of Extremadura, 06006 Badajoz, Spain; joanapoejo86@gmail.com (J.P.); garcialopez@unex.es (V.G.-L.); virginio@unex.es (V.G.-M.); 2 Department of Human Anatomy and Embryology, Faculty of Medicine and Health Sciences, University of Extremadura, 06006 Badajoz, Spain; 3 School of Technology and Management, Polytechnic Institute of Leiria, Morro do Lena-Alto do Vieiro, 2411-901 Leiria, Portugal; ricardo.lagoa@ipleiria.pt; 4 Laboratory of Separation and Reaction Engineering-Laboratory of Catalysis and Materials (LSRE-LCM), Polytechnic Institute of Leiria, 2411-901 Leiria, Portugal; 5 Department of Medical and Surgical Therapeutics, Pharmacology Area, Faculty of Medicine and Health Sciences, University of Extremadura, 06006 Badajoz, Spain

**Keywords:** kaempferol, flavonoids, brain stroke, ischemia reperfusion, 3-nitropropionic acid, Huntington’s disease, brain neurodegeneration, pharmacological implications

## Abstract

Kaempferol, a flavonoid present in many food products, has chemical and cellular antioxidant properties that are beneficial for protection against the oxidative stress caused by reactive oxygen and nitrogen species. Kaempferol administration to model experimental animals can provide extensive protection against brain damage of the *striatum* and proximal cortical areas induced by transient brain cerebral ischemic stroke and by 3-nitropropionic acid. This article is an updated review of the molecular and cellular mechanisms of protection by kaempferol administration against brain damage induced by these insults, integrated with an overview of the contributions of the work performed in our laboratories during the past years. Kaempferol administration at doses that prevent neurological dysfunctions inhibit the critical molecular events that underlie the initial and delayed brain damage induced by ischemic stroke and by 3-nitropropionic acid. It is highlighted that the protection afforded by kaempferol against the initial mitochondrial dysfunction can largely account for its protection against the reported delayed spreading of brain damage, which can develop from many hours to several days. This allows us to conclude that kaempferol administration can be beneficial not only in preventive treatments, but also in post-insult therapeutic treatments.

## 1. Introduction

**Figure 1:**
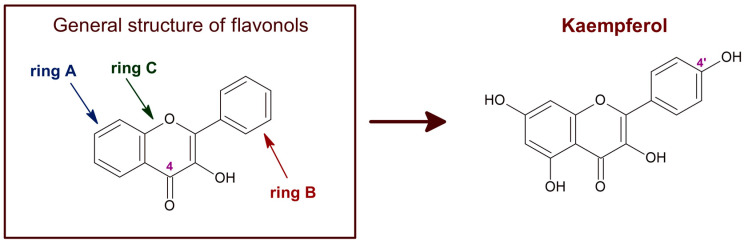
Chemical structure of flavonols in general and of kaempferol. The C4 position of the characteristic carbonyl group of flavonols is marked. Kaempferol, also known as 3,4′,5,7-tetrahydroxyflavone [chemical name: 3,5,7-trihydroxy-2-(4-hydroxyphenyl)-4H-1-benzopyran-4-one], bears a single hydroxyl group in ring B, at position 4′.

Kaempferol is a polyphenol of the flavonol family of flavonoids. Therefore, kaempferol’s chemical structure (Figure 1) is closely related to other flavonols with beneficial chemotherapeutic properties, like quercetin [1,2]. Kaempferol, like other flavonoids, is largely present as a glycoside derivative in plants. Apart from the most abundant glycoside derivatives, kaempferol-3-O-glucoside (astragalin), kaempferol-3,7-dirhamnoside (kaempferitrin) and kaempferol-3-O-rutinoside (nicotiflorin) [3], many other bioactive kaempferol glycosides have been found in plants, like kaempferol 3-O-[(6-O-E-caffeoyl) β-D-glucopyranosyl-(1→2)]-β-D-galactopyranoside-7-O-β-D-glucuropyranoside, kaempferol 3-O-[(6-O-E-p-coumaroyl)-β-D-glucopyranosyl-(1→2)]-β-D-galactopyranoside-7-O-β-D-glucuropyranoside and kaempferol 3-O-[(6-O-E-feruloyl)-β-D-glucopyranosyl-(1→2)]-β-D-galactopyranoside-7-O-β-D-glucuropyranoside [4,5].

Kaempferol and its glycoside derivatives can be found in a variety of plants and plant-derived food products, such as spinach, kale, cabbage, chives, lentils, tea, broccoli, apples, and *Ginkgo biloba*, among others [5,6,7,8,9]. The plasma concentrations of total flavonoid metabolites vary from 0 to 2.0 μM, with an intake of 50 mg aglycone equivalents in humans, although isoflavones can reach higher concentrations, as well as metabolites generated by the gut [10,11]. After food intake, bacterial enzymes and enterocyte β-glucosidases of the intestine hydrolyze flavonoid glycosides to their aglycones [12,13]. In addition, flavonoid aglycones undergo methylation and glucuronidation in the intestine [14] and their metabolites are further metabolized by the liver [12]. It should be noted that glial cells can also catalyze in vitro oxidation and conjugation of flavonoids with reduced glutathione (GSH) [15]. Although flavonoid metabolites are still bioactive as cellular antioxidants, most of these metabolites have been shown to have lower chemical antioxidant potency than the aglycone flavonoid [16,17,18], and this also seems to be the case of kaempferol [19]. Furthermore, the kaempferol glycosides most abundant in plants (astragalin, kaempferitrin, and nicotiflorin) have poorer antiproliferative activity than kaempferol [20]. Therefore, the medicinal use of kaempferol requires us to pay special attention to the administration route. Also, if the concentration of blood kaempferol needed in therapeutic treatments of brain damage is higher than 1–2 μM, instead of oral supplementation, other methods of administration must be used, i.e., intravenous (IV) or microencapsulation delivery.

The redox potential of kaempferol, ~0.39 V [21,22], indicates that this compound is a chemical antioxidant. Furthermore, kaempferol, as well as other flavonoids, can form stable radicals that may act as reactive species scavengers [23]. The acceptance of flavonoids as cellular antioxidants is largely based upon their ability to scavenge hydroxyl radicals, superoxide anion, lipid peroxides, and peroxynitrite [23,24,25,26,27,28]. The number of hydroxyl groups in the B-ring and the presence of a carbonyl group in C4 of the C-ring are particularly relevant for the scavenging of these reactive oxygen species (ROS) [27]. Of note, the flavonoids that most efficiently scavenge hydroxyl radicals are flavonols [23], i.e., the family of flavonoids to which kaempferol belongs (Figure 1). Moreover, compared with other phenols and flavonoids, kaempferol has a relatively high peroxynitrite scavenger activity [29,30]. Another property of flavonoids relevant for their role as cellular antioxidants is their liposolubility. The inhibition of lipid peroxidation by flavonoids correlates with their partition coefficient between n-octanol and water, because flavonoids need to incorporate into the lipid bilayer to trap the species initiating lipid oxidation radical chains [31,32]. Indeed, flavonoids act as hydrogen donors in the reaction with the peroxyl radical produced in the oxidation of fatty acids [33]. It has been shown that the higher solubility in water can account for the lower potency of flavonoid glycosides as inhibitors of lipid peroxidation compared with their respective flavonoid aglycones [34,35]. In addition, the solubility of flavonoids in lipid bilayers favors its uptake by cells and tissues. Notably, kaempferol is one of the flavonoids with a higher lipid/water partition coefficient [36].

After feeding rats with an alcoholic extract of *Hypericum perforatum* (St. John’s wort) or pure isoquercitrin (quercetin glycoside), flavonols (like quercetin and their metabolites isorhamnetin/tamarixetin) have been detected in brain homogenates [37]. The ability of kaempferol to overcome the blood–brain barrier has been shown after administration of a single dose of 600 mg/kilograms (kg) in rats [38]. Moreover, the brain accessibility to flavonoids should be increased upon blood–brain barrier disruption that has been reported in all neurodegenerative disorders [39]. Indeed, the many protective actions of kaempferol against brain diseases and insults point out that this flavonoid and its derived metabolites are readily accessible to the ill-brain. Also, the IV administration of flavonoids is expected to improve the brain’s accessibility of flavonoids.

The aim of this article is to perform an overview of the main results and conclusions achieved in our previous works with kaempferol [1,2,40,41,42,43,44,45,46,47,48], which are presented within the context of the results and findings reported up to date in publications of other investigators. To this end, in the next sections of this review we shall address the following points: kaempferol administration efficiently prevents brain damage induced by ischemic insults (Section 2); kaempferol administration efficiently prevents brain damage induced by 3-nitropropionic acid (Section 3); molecular and cellular mechanisms that contribute to kaempferol protection against the brain damage produced by ischemia-reperfusion and by NPA administration (Section 4); and short integrative conclusions (Section 5).

## 2. Kaempferol Administration Efficiently Prevents Brain Damage Induced by Ischemic Insults

The brain ischemic insult elicited by a stroke episode is a major health problem in humans, as they often cause death or chronic disability. Ischemia stroke is the most frequent type of cerebrovascular stroke [49,50]. The transient blockade of blood flow produces rapid tissue damage in the so-called ischemic core. The rapid metabolic energy falls, leading to the depolarization of cell membranes, which causes rapid necrosis in the ischemic core. Later, the brain damage extends into the peri-infarct region [51]. Restoration of blood flow (reperfusion) is necessary, but this can aggravate brain damage [52,53], as it leads to disability and a high morbidity in patients due to irreversible brain damage. The excessive production of ROS and reactive nitrogen species, excitotoxicity, and brain inflammation are implicated in the neuronal damage during a brain ischemia-reperfusion injury [54]. Of note, oxidative stress-mediated inflammation and apoptosis are considered to play a crucial role in brain damage associated with ischemia-reperfusion episodes [55,56]. Glutamate excitotoxicity [51,57,58], oxidative stress [51,59,60], edema and inflammation [51,58], activation of matrix metalloproteinases [61,62,63,64], and apoptosis [60,65,66] contribute to spreading the brain damage to the peri-infarct region. The slow time course of this phase opens a temporal window for neuroprotective interventions.

Protective effects of different flavonoids against the ischemic brain damage produced by cerebral ischemia have been shown in many studies with experimental animals, reviewed in [1]. As noted in [1], these studies showed that the efficiency of protection against brain damage in transient cerebral ischemia largely varies between different flavonoids and depends on the administration route and doses. For example, the neuroprotective action of *Ginkgo biloba* extract EGb761, which is rich in kaempferol, in brain ischemia models seems to be dependent on the expression of heme oxigenase-1 [67], an enzyme that is modulated by kaempferol in different cells [68,69]. Also, nicotiflorin (kaempferol-3-O-rutinoside) has neuroprotective effects in permanent [70] and transient [71] models of rat cerebral ischemia.

**Figure 2:**
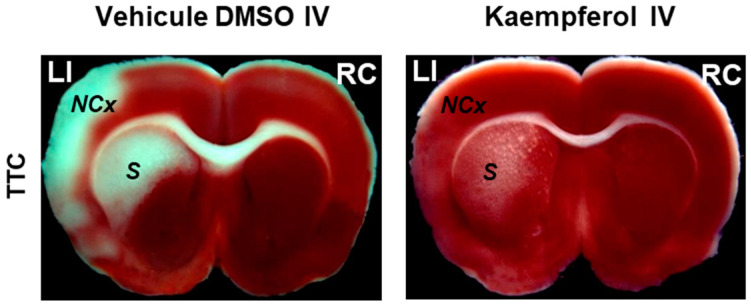
Kaempferol protects against ischemia/reperfusion-induced brain damage. The images of brain slices stained with 2,3,5-triphenyltetrazolium chloride (TTC) show the reduction of the damage extension in brain areas (NCx: neocortex and *S: striatum*) after kaempferol treatment in rats subjected to ischemia/reperfusion. LI and RC mean left ischemic and right control hemispheres, respectively. Meaning of other abbreviations used in this figure: DMSO (dimethyl sulfoxide) and IV (intravenous administration).

**Figure 3:**
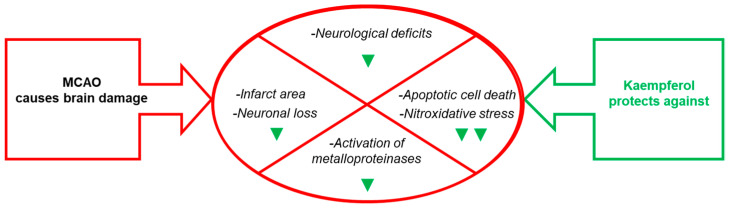
Effects of kaempferol administration highlighted in this section. Kaempferol reduces MCAO- induced brain damage in the infarct area, neurological deficits, apoptotic cell death and neuronal loss, as well as the levels of biomarkers of brain damage. The green symbols indicate attenuation by kaempferol: ▼ and ▼▼ mean partial and nearly complete attenuation, respectively. See the text for further details.

To study the effect of the IV administration of kaempferol on brain damage induced by ischemia-reperfusion, first we setup a rat model of transient focal ischemia using a new surgical technique of middle cerebral artery occlusion (MCAO) by selective endovascular placement of a guide-wire [72]. Our study published in 2007 [41] has been, to the best of our knowledge, the first work that reported on the role of kaempferol administration in the protection against the brain damage caused by transient cerebral ischemia. The main results and conclusions obtained in this work are summarized next (Figure 2 and Figure 3).

Our rat model of transient focal ischemia-reperfusion presented brain damage largely focalized in the *striatum*, *hippocampus*, and closer areas of the neocortex. This result was in good agreement with the brain areas that were reported to be more sensitive to this insult in earlier works [64,73]. We found that IV injections of kaempferol through a catheter inserted into the tail vein afforded very efficient protection against rat brain damage [41,42]. Later, Yu et al. [74] reported that IV administration of kaempferol-3-O-rhamnoside and kaempferol-3-O-glucoside attenuated neurological deficits caused by ischemia, preventing neuron and axon damage in an in vivo MCAO-induced ischemic stroke model. The protocol used in [41] was one IV injection 30 min before the induction of focal cerebral ischemia and a second injection just before starting the reperfusion. After testing the effects of 0.7 mL IV injections of different concentrations of kaempferol, we concluded that blood kaempferol concentrations between 10 and 15 μM afforded an almost complete protection against rat brain infarct by transient cerebral ischemia induced by middle cerebral artery occlusion. Moreover, we estimated that a blood concentration of 3–4 μM of kaempferol produced half of the maximum protection effect. Since oral ingestion reaches micromolar concentrations of flavonoids in the bloodstream [75], this latter result allowed us to suggest that “diet- or pharmaceuticals-supplementation with this flavonoid may be helpful to attenuate ischemic/reperfusion-induced brain damage” [41]. Indeed, in a recent work, Zhang et al. [76] have reported that intragastric kaempferol administration reduces the infarct volume and improved neurological function after transient focal cerebral ischemia-reperfusion injury in adult male Sprague–Dawley rats. Also, beneficial neuroprotective effects against ischemic stroke in other MCAO models have been reported during recent years for oral administration of kaempferol to mice [77], and for intragastric administration to rats of kaempferol [78], and of kaempferide-7-O-(4″-O-acetylrhamnosyl)-3-O-rutinoside [79]. The reported low toxicity of flavonoids to humans [80,81] and their slow renal clearance in mammals [80,81,82] are also factors that favor its pharmaceutical use.

Our work published in [41,42] also led to the conclusion that IV injections of kaempferol produce an almost complete protection against cell death through apoptosis in transient cerebral ischemia induced in the ischemic hemisphere by middle cerebral artery occlusion in the temporal-frontal areas of the neocortex and *striatum*. Later on, Wang et al. [79] showed that intragastric administration of the kaempferol-related flavonoid kaempferide-7-O-(4″-O-acetylrhamnosyl)-3-O-rutinoside decreased neurological and histological deficits, reduced the infarct volume, and decreased neuroapoptosis in another MCAO rat model. More recently, Zhang et al. [76] have shown that intragastric kaempferol administration also attenuated cell apoptosis in cerebral ischemia-reperfusion after transient focal cerebral ischemia-reperfusion injury in adult male Sprague–Dawley rats. In [41], this conclusion was experimentally supported by the >90% reduction of terminal deoxyribonucleotidyl transferase-mediated dUTP-fluorescein nick-end labeling (TUNEL-histochemistry), >90% reduction of the increase of poly-ADP polymerase degradation and 80–90% reduction of caspase-9 activity in the ischemic brain hemisphere. Also, in [41] it was noted that the efficiency of protection afforded by kaempferol treatment was lower, i.e., 40–50%, in the hippocampus and vicinal caudal areas of the basal ganglia located in coronal slices 8–10 mm away from the front pole of the rat brain. Notably, kaempferol has been reported to have a limited ability to protect against excitotoxic neuronal death [83], and glutamate-excitotoxic neuronal death plays a significant role in the death of hippocampal neurons induced by ischemia/reperfusion [84,85]. This might be a plausible cause accounting for the large, but not complete, attenuation by kaempferol IV administration of the ischemia-reperfusion-induced activation of matrix metalloproteinases, which displays a regional pattern similar to that found with 2,3,5-triphenyltetrazolium chloride (TTC) staining [41].

In addition, the results reported in [41,42] pointed out that apoptotic cell death is the major cell death pathway in the neocortex and *striatum* after the transient focal cerebral ischemia insult, because the values obtained for attenuation by kaempferol of TTC staining are only slightly higher than those obtained from attenuation of TUNEL staining. Therefore, we concluded that the IV injections of kaempferol applied in this work can be very efficient in protecting against rat brain damage through apoptosis after transient focal cerebral ischemia-reperfusion. Recently, it has been shown that intragastric kaempferol administration inhibits the expression of pro-apoptotic proteins and promotes the expression of anti-apoptotic proteins at doses that significantly reduce the volume of cerebral infarction and the neurological deficits after transient focal cerebral ischemia-reperfusion injury in adult male Sprague–Dawley rats [76]. Owing to the leading role of apoptosis in delayed death after ischemia-reperfusion [86], the attenuation of apoptosis afforded by kaempferol highlights its high value as a neuroprotective agent to limit brain damage after acute cerebral ischemia. Notably, in a previous work we showed that kaempferol efficiently protects against the oxidative-stress mediated apoptosis of rat cerebellar granule neurons in culture [40], a widely used model for neuronal apoptosis [87,88]. Interestingly, the concentration of kaempferol that affords 50% inhibition of the rat cerebellar granule neurons apoptosis, 8 ± 2 μM [40], is within the blood concentration range of kaempferol affording a large protection against the brain damage induced after transient focal cerebral ischemia/reperfusion in the rat model used in [41]. Consistent with kaempferol protection against oxidative-stress mediated apoptosis, in [41] it is shown that IV injections of kaempferol also blocks the increase of protein nitrotyrosines in the ischemic hemisphere. Therefore, IV injections of kaempferol can provide a potent antioxidant protection against nitric oxide-derived radicals in the brain, which have been shown to be generated in ischemia-reperfusion brain insults [51]. In addition, these results give a strong support to a leading role for peroxynitrite-mediated oxidative stress in the apoptotic cell death observed in the brains of our rat model of transient focal cerebral ischemia-reperfusion. It must be recalled here that other investigators showed earlier that peroxynitrite and other reactive nitrogen species play a major role in brain damage associated with ischemia-reperfusion [51,89].

## 3. Kaempferol Administration Efficiently Prevents Brain Damage Induced by 3-Nitropropionic Acid

The 3-nitropropionic acid (NPA) is a neurotoxin for cattle and humans and is produced by some fungi and plants [90,91]. This neurotoxin can induce brain *striatum* degeneration and neurological disturbances that mimic some aspects of Huntington’s disease (HD) when administered systemically to rodents and non-human primates [92,93,94,95]. The systemic administration of NPA also produces metabolic alterations in cortical areas adjacent to the *striatum*, as well as in the *hippocampus* and the *cerebellum* [96,97], which can account for neurological alterations seen in pre-motor stages of HD, like cognitive dysfunction, visuospatial deficits, memory loss, and difficulty in learning new skills [98,99].

NPA is a suicide inhibitor of succinate dehydrogenase [100,101,102] and causes the rapid loss of ATP in cultured neurons in vitro [103,104]. The enhanced generation of ROS that activate cell death pathways due to the impairment of mitochondrial function has been accepted to play a major role in the neurotoxicity of NPA [94,104,105,106,107,108,109]. The relevance of NPA-induced striatal degeneration models as animal models of HD is further supported by reported anomalies in mitochondrial function and oxidative stress in the brain degeneration of HD patients [106,110].

**Figure 4:**
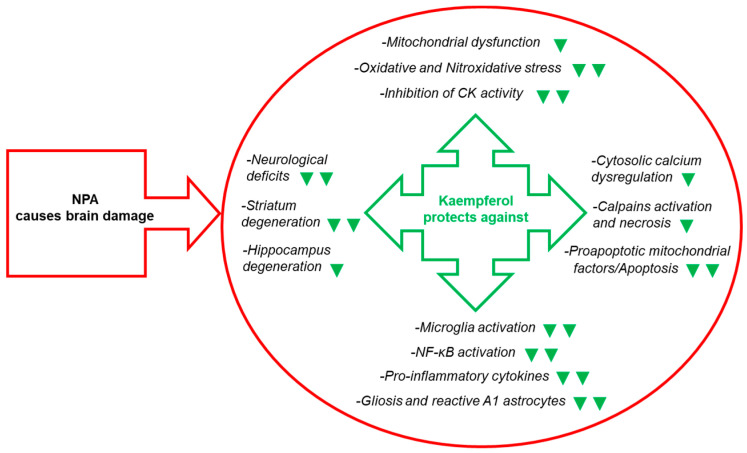
Kaempferol reduces NPA-induced brain lesion, neurological dysfunctions, and, also, the levels of many biochemical and cellular biomarkers of brain degeneration. The green symbols indicate attenuation by kaempferol of the NPA-induced change: ▼ and ▼▼ mean partial and nearly complete attenuation, respectively. Abbreviations used in this figure: CK (creatine kinase) and NF-κB (nuclear factor kappa light-chain enhancer of activated B cells). See the text for further details.

To the best of our knowledge, our work [43] was the first report of kaempferol protection against NPA-induced brain damage and associated neurological dysfunctions in an animal model. In this work we used chronic treatment of Wistar rats with intraperitoneal (IP) injections of 25 mg of NPA/kg of body weight (BW) in normal saline (0.9% *w*/*v* NaCl) every 12 h during several days. We assessed that this treatment induced neurological disturbances (hypoactivity, dystonic movements of hind limbs, and an abnormal gait) and striatal degeneration similar to those reported in previous publications of other investigators [92,95]. We showed that IP injections of 21 mg of kaempferol/kg BW 48 h before the first NPA treatment and every day 30 min prior to the morning NPA injection largely attenuated NPA-induced neurological disturbances [43,44]. Also, our results pointed out an NPA-induced lesion highly localized in the *striatum*, because all the histological markers used (TTC, hematoxylin-eosin and TUNEL staining) did not show a significant increase in the vicinal brain cortical area, and IP daily doses of 21 mg of kaempferol/kg BW afforded an almost complete blockade of the striatal lesion (Figure 4).

The chronic treatment of rats with NPA produces inhibition of the succinate dehydrogenase activity of the mitochondrial fraction of rat striatal lysates [43,100,101,102]. However, our results demonstrated that IP injections of up to 21 mg of kaempferol/kg BW did not prevent the inhibition of the succinate dehydrogenase activity in the *striatum* [43]. Since creatine administration have been shown to afford neuroprotection against NPA-induced degeneration in rats [111] and beneficial effects in HD patients [112,113], we evaluated the effect of the NPA treatment on the creatine kinase activity in our experimental rat model. Our results also pointed out a large inhibition of the activity of both cytosolic and mitochondrial creatine kinase isoforms in the *striatum* of NPA-treated rats and, also, that IP injections of up to 21 mg of kaempferol/kg BW fully prevented the inhibition of the creatine kinase activity in the *striatum* [43]. It must be recalled that creatine kinase is the major cytosolic ATP buffering system in neurons, and its inhibition should lead to a large, rapid and sustained fall of cytosolic ATP, particularly in neurons where the mitochondrial respiration is impaired by NPA. Moreover, creatine kinase has been proposed to be a target of oxidative stress in HD patients using brain *pos-tmortem* samples [114], and a marked reduction of creatine kinase activity has been reported in the brain of patients with oxidative stress-linked neurodegenerative diseases, such as Alzheimer’s, Parkinson’s and Pick’s diseases [115,116,117]. As noted in [43], the inhibition of cytosolic creatine kinase activity correlated with a large increase of protein nitrotyrosines and, also, with the large decrease of the level of this protein in striatal lysates, which is consistent with the enhanced susceptibility of oxidized proteins to undergo proteolytic degradation [118]. Thus, the NPA-induced fall of creatine kinase activity in brain neurons is likely to be one of the more significant biochemical mechanisms underlying the bioenergetic crisis associated with NPA neurotoxicity.

On the other hand, a sustained ATP fall in the neuronal cytosol leads to a rapid necrotic cell death, which is preceded by a sustained rise of cytosolic calcium up to the neurotoxic range. Note that P-ATPases play a major role in resetting the Na^+^, K^+^ and Ca^2+^ gradients in neurons during normal activity and, also, in maintaining the cytosolic calcium within the narrow concentration range required for neuronal survival [119]. In turn, neuronal nitric oxide synthase (nNOS) stimulation by a sustained rise of neuronal cytosolic calcium generates an overshot of nitric oxide production, which results in a large nitroxidative stress and produces highly neurotoxic oxidants like peroxynitrite and hydrogen peroxide [52,117,120,121,122]. The loss of cytosolic calcium homeostasis in neurons is further accelerated by these strong oxidants through oxidative modifications of the major transport systems in control of neuronal calcium homeostasis [123,124]. Nitric oxide-derived oxidants play a major role in NPA-induced brain damage, because aminoguanidine, an inducible nitric oxide synthase (iNOS) inhibitor, attenuated NPA neurotoxicity [125], and L-N^G^-nitro arginine methyl ester, a widely used inhibitor of NOS, reduces the striatal lesion volume induced by NPA [126]. Indeed, iNOS immunolabelling was observed in the *striatum* of NPA-treated rats [125], and increased levels of endothelial nitric oxide synthase (eNOS) mRNA in the rat *striatum* have been reported after treatment with NPA for 2 days [127]. Furthermore, an excess of ROS and nitric oxide potentiates NPA neurotoxicity because it causes both reversible and irreversible damage to the mitochondrial respiratory chain function [128]. In our rat model, we found a large increase of protein nitrotyrosines in rat striatal lysates induced by NPA treatment, more than eight-fold increase over the basal level in saline-injected rats, and only a moderate increase, lower than two-fold increase, in proximal cortical areas [43,44]. The treatment of rats with IP injections of 21 mg of kaempferol/kg completely blocked the NPA-induced increase of protein nitrotyrosines. However, the oxidative stress induced in the *striatum* by the treatment with NPA is not only nitroxidative stress, as antioxidants like the glutathione precursor *N*-acetylcysteine [129], S-allylcysteine [130], coenzyme Q10 [131], and vitamin E [109] have been reported to protect rat or mouse against NPA-induced neurodegeneration. Indeed, we also found that this dose of kaempferol completely protects against the large decrease of the content of reduced glutathione in the striatal lysates elicited by the treatment solely with NPA [43,44]. Therefore, a 21 mg/kg dose of kaempferol works as a highly efficient antioxidant against the large oxidative stress mediating NPA-induced damage in the rat *striatum*.

A sustained rise of cytosolic calcium should strongly stimulate calpains, whose activation has been shown to be sufficient to produce a rapid irreversible evolution towards neuronal necrotic death [104,132,133,134]. Indeed, it has been shown that calpains activation mediates NPA-induced neurotoxicity to neuronal cultures in vitro [104,135,136], and also in experimental rat models [137,138]. We observed a near two-fold stimulation of the calpain activity in striatal lysates prepared from NPA-treated rats with respect to control rats, a stimulation that was blocked with 14 or 21 mg/kg BW doses of kaempferol [43,44]. Moreover, the treatment with 14 or 21 mg/kg BW doses of kaempferol efficiently prevented the accelerated proteolysis underlying the overall protein loss, suggesting that kaempferol could be blocking other proteases activated by the NPA treatment as well.

Neuroinflammation can contribute to spreading an initially focalized neuronal insult to different types of brain cells and structures, and NPA administration induces the activation of neuroinflammatory microglia [139,140,141], which has also been reported in the *striatum* and proximal cortical areas in HD patients [142]. Activated microglia enhances ROS and nitric oxide production in the brain and also secretes pro-inflammatory cytokines [143]. Furthermore, the oxidative stress caused by mitochondrial dysfunction activates the nuclear factor kappa light-chain enhancer of activated B cells (NF-κB) signaling pathway, leading to enhanced secretion of the proinflammatory cytokines that mediate NPA-induced brain degeneration [139,140,141]. Moreover, cathepsins, which are activated in brain neurodegenerative processes [144,145], can act as auxiliary proteases in the proteolytic processing of C3 [146], which is an accepted biomarker of neurotoxic A1 astrocytes abundant in *post-mortem* tissue of HD patients [147]. Rats treated with systemic administration of NPA show astrocytes dysfunctions or gliosis in the *striatum* [43,47,141,148,149]. The rise in C3α levels is an early event in NPA-induced neurodegeneration of the rat brain, as it precedes the appearance of severe neurological motor dysfunctions [47]. Of note, the induction of reactive A1 astrocytes by IP NPA administration in the *hippocampus* [47] may help to rationalize the memory impairment produced by systemic NPA administration to rodents [97,150]. As shown in [48], the activation of complement C3 protein and generation of reactive A1 astrocytes induced by acute treatment with NPA in the *striatum* and the *hippocampus* is efficiently prevented by IP daily doses of 21 mg of kaempferol/kg BW The IP administration of these doses of kaempferol results in an almost complete blockade of the NPA-induced increase of C3α and other proteolytic fragments of C3 (iC3b, C3α fragment 2, and lower molecular weight fragments) in the *striatum* and *hippocampus*. Let us recall here that this IP administration of kaempferol also affords an almost complete protection against the neurological motor dysfunctions and against the rise of markers of brain damage induced by acute IP injections of the neurotoxin NPA in male adult Wistar rats [43,48]. Notably, herbal extracts of *Persicaria lapathifolia* that contain kaempferol glycoside have been shown to inhibit the classical pathway of complement C3 protein activation [151]. In addition, the results reported in [48] indicate that kaempferol also prevented microglia activation in NPA-induced brain degeneration.

The histochemical results lend strong support to the large therapeutic potential of kaempferol for protection against NPA-induced brain degeneration in the major brain regions affected by this neurotoxin, since the levels of tissue markers of NPA-induced brain neuroinflammation (C3 activation, NF-κB immunostaining, astrogliosis, pro-inflammatory cytokines interleukin-1α and tumor necrosis factor α, and C1q) and neurodegeneration (TTC staining and TUNEL labeling), in *striatum* and *hippocampus* slices from rats of the group treated with kaempferol and NPA are similar to those found in the slices of these brain areas from the control group [43,48]. Also, it must be noted that the effective doses of kaempferol that afford protection against brain damage are strongly dependent on the administration route of this flavonoid. For comparison, IV injections of 0.16–0.25 mg of kaempferol/kg BW produce extensive protection against the striatal neurodegeneration caused by transient focal cerebral ischemia induced by middle cerebral artery occlusion in adult rats [41,42], while IP injections reported to reach a similar level of protection against NPA-induced brain damage are 14–21 mg of kaempferol/kg BW [43,48].

## 4. Molecular and Cellular Mechanisms That Contribute to Kaempferol Protection against the Brain Damage Produced by Ischemia-Reperfusion and by NPA Administration

**Figure 5:**
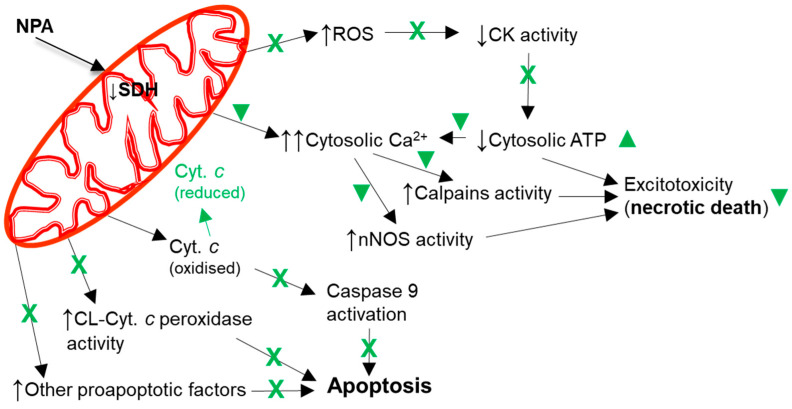
Kaempferol protects against mitochondrial dysfunctions leading to cellular bioenergetics crisis and apoptosis. Black arrows indicate the effects of NPA administration. The reduction of cytochrome *c* by kaempferol is highlighted in green. A green cross (**X**) means almost complete protection and a green symbol (▼) means partial protection. Abbreviations used in this figure: CK (creatine kinase), CL-Cyt. *c* peroxidase (cardiolipin-induced cytochrome *c* peroxidase activity), Cyt. *c* (cytochrome *c*), and SDH (succinate dehydrogenase). See the text for further information.

A mitochondrial energetics failure is the initial event in the brain degeneration induced by both ischemic injury (oxygen supply shortage) and NPA administration (inhibition of mitochondrial succinate dehydrogenase) (Figure 5). Notably, in vitro studies have shown that flavonoids can afford an effective protection in the 1–10 μM range in cell death models where mitochondrial dysfunction and apoptosis are implicated [40,152,153,154,155]. More recently, it has been noted that the beneficial effects of kaempferol after traumatic injury in the rats developing brain (a model system used for understanding the molecular mechanisms underlying the distinct neuropathological consequences of traumatic brain injury in children) is through protection of mitochondrial function, oxidative metabolism, and neural viability [156]. As shown in [45], kaempferol was one of the most potent flavonoids as an inhibitor of the rate of H_2_O_2_ production by brain mitochondria, which reaches nearly 100% inhibition with 10 μM flavonoid with 50% inhibition concentration of 1.8 μM. However, up to 10 μM kaempferol did not significantly affect the oxygen consumption rate of brain mitochondria [45,153]. The major systems involved in ROS production by respiring mitochondria are mitochondrial respiratory complexes I and III [157,158]. In [45], we identified the mitochondrial respiratory complex I as the molecular target of kaempferol for the inhibition of mitochondrial ROS production, and found kinetic competition between CoQ and kaempferol modulation of the activity of complex I, suggesting that kaempferol binds to complex I at a site, at least, partially overlapping with the quinone-inhibitor binding pocket. It should be recalled here that mitochondrial respiratory complex I has been proposed to be the source of ROS in models of heart failure and, also, the initial site of ischemia-elicited damage to mitochondria in the heart [159]. In addition, an impaired function of the mitochondrial respiratory complex I is well documented in several neurodegenerative diseases [157]. Notably, oral kaempferol administration decreases mitochondrial fission and helps to preserve mitochondrial functional integrity and morphology in a C57BL/6 mice MCAO model of ischemic stroke [77].

Mitochondrial dysfunction also plays a major role in apoptotic cell death, which has been shown to be strongly attenuated by kaempferol administration to animal models of ischemia-reperfusion and NPA-induced brain degeneration, as indicated in the previous Section 2 and Section 3. Indeed, it has been shown that kaempferol administration can inhibit the expression of pro-apoptotic proteins and promote the expression of anti-apoptotic proteins in animal models of ischemia-reperfusion [76,79]. Furthermore, the IP administration of kaempferol inhibits apoptosis induced by NPA in the *striatum* [43], and the IV administration of kaempferol in a rat MCAO model inhibits apoptosis and caspase-9 activity in the *striatum* [41]. Similar results have been reported later with the administration of kaempferide-7-O-(4″-O-acetylrhamnosyl)-3-O-rutinoside in a MCAO ischemic model [79]. Cytochrome *c* has a key role in the initial stages of the apoptotic pathway linked to mitochondrial dysfunction (Figure 5), because cytochrome *c* can trigger apoptosome assembly and subsequent activation of caspases executing the programmed cell death [160,161,162,163]. However, reduced cytochrome *c* has little or no capacity to activate the caspases in the apoptosome [162,164], enabling the regulation of the apoptosis by modulation of cytochrome *c* redox state [46,162,165]. Indeed, it has been shown in cellular and in heart ischemia models that several efficient reductants of cytochrome *c* prevent caspase activation and apoptosis [164,166,167]. We have demonstrated that kaempferol reduces cytochrome *c* and that this is a fast chemical reaction [2]. Moreover, we showed that kaempferol inhibition of the cardiolipin-induced peroxidase activity of cytochrome *c* correlates with its potency to reduce cytochrome *c*. The binding of cytochrome *c* to cardiolipin, a lipid highly enriched in mitochondrial membranes, activates its peroxidase activity caused by cytochrome *c* partial unfolding that increase the accessibility of the heme group to small molecules such as H_2_O_2_ [168,169,170,171], and a decrease in the cytochrome *c* redox potential [169,172,173,174,175]. The activation of the cytochrome *c* peroxidase activity by cardiolipin has been shown to be a very early event in the intrinsic apoptotic program and triggers cytochrome *c* release from the mitochondria to the cytosol [172,176], likely through nanoscale pores in cardiolipin-containing membranes [177]. On these grounds, we proposed in [2] that the reduction of cytochrome *c* and inhibition of its cardiolipin-induced peroxidase activity by kaempferol are likely contributing to the slowdown and eventual blockade of the mitochondrial-pathway leading to intrinsic apoptosis before reaching the irreversible stages triggered by the activation of caspases 3 and 9. It must be noted here that the activation of caspases is detectable only hours after the brain insult induced either by ischemia-reperfusion in MCAO or NPA-treated animal models.

In addition, as dysfunctional mitochondria are a major source of ROS in mammalian cells, the antioxidant properties of kaempferol are also expected to contribute to its protective action against the brain damage induced both by ischemic-reperfusion injury and by NPA intoxication. Nevertheless, it must be noted that the flavonoids potency as cellular antioxidants cannot be accounted solely in terms of their chemical antioxidant capacity, as there is a lack of correlation between the reduction potential of flavonoids and their capacity to protect against oxidative neuronal death induced by different insults in model neuronal cultures [1,83,178,179]. In addition, the protective effective concentrations of the flavonoids do not correlate with their differential free radical scavenging capacity [180,181,182]. Indeed, different laboratories have shown that flavonoids attenuate cellular ROS production through inhibition of many redox enzymes, such as NAD(P)H oxidases, xanthine oxidase, monooxygenases, cyclooxygenase and lipoxygenases [40,183,184,185,186,187,188,189]. Also, the iron-complexation by flavonoids lends protection against the harmful Fenton reactions and free radical chain reactions in the cells, like Fe^2+^-induced lipid peroxidation [6,27,80,185,190,191]. Kaempferol is one of the flavonoids that more strongly inhibits Fe^2+^-induced lipid peroxidation and, also, reduces the ferrylmyoglobin radical, a harmful radical generated in cardiac ischemic reperfusion upon reaction of metahemoglobin with hydrogen peroxide [6,80,191,192].

Owing to the relevant role of excitotoxicity in the brain damage caused by ischemia-reperfusion injury (Section 2) and, also, by NPA administration (Section 3), it bears particular relevance here the ability of polyphenolic antioxidants to block the activation of calpains in neuronal cultures in vitro, thereby affording neuroprotection against excitotoxic death [193]. An important role of N-methyl-D-aspartate receptors in NPA neurotoxicity has been shown in several publications [103,194,195,196]. Akashiba et al. [138] have shown the stimulation of N-methyl-D-aspartate receptors in striatal and cortical neurons death induced by neurotoxic concentrations of NPA. Recently, Lin et al. [197] have shown that micromolar concentrations of kaempferol 3-rhamnoside inhibit glutamate release in isolated rat cerebrocortical nerve terminals. Calabresi et al. [136] pointed out that this stimulation is observed in striatal medium-sized spiny neurons for which NPA is very toxic, but not in other neurons known to be resistant to NPA toxicity. Since the maintenance of cytosolic Ca^2+^ homeostasis in stimulated glutamatergic neurons critically depends on the activity of Ca^2+^-pumps (Ca^2+^-ATPases) [119], the protection by kaempferol against creatine kinase inhibition (Figure 5), analyzed in more detail in Section 3, should enhance the resistance of neurons against excitotoxic cell death, as it will delay the fall of cytosolic ATP. Kaempferol can also contribute to attenuate calpain activation either through the stimulation of mitochondrial calcium uniporter which helps to maintain cytosolic Ca^2+^ homeostasis [198], or through the maintenance of mitochondrial membrane potential and prevention of delayed calcium deregulation [153].

The sustained rise of cytosolic Ca^2+^ concentration leads to a nitric oxide overshot within the neurons undergoing glutamate-induced excitotoxic neuronal death (Figure 5), due to the stimulation of nNOS [119]. Despite the fact that nitric oxide is a diffusible molecule across the lipid membranes and, therefore, can reach vicinal cells, the activation of microglia and gliosis affords the major contribution to the spreading of an initially focalized brain damage. This is also a delayed event in brain degeneration induced either by ischemia-reperfusion in MCAO or NPA-treated animal models, as it is detected only days after the initial brain insult. Brain inflammation has been shown to play a causal role in the neurodegeneration in ischemia-reperfusion injury (Section 2) and in NPA intoxication (Section 3), and recent reviews have highlighted the molecular mechanisms of action of flavonoids as anti-inflammatory molecules, see, for example [199,200]. The anti-inflammatory actions of kaempferol are well recognized [201,202], and it is to be noted that recently it has been shown with lipopolysaccharide-stimulated BV2 microglial cells that its derived glucuronate metabolite also displays cellular antioxidant and anti-neuroinflammatory activity [203]. Kaempferol administration has been shown to inhibit astrogliosis and/or microglia activation in the brain of animal models of MCAO [41,76,78,79] and of NPA-induced neurological dysfunction [43,48,139,140,141]. The down-regulation of proinflammatory cytokines gene expression by kaempferol and related flavonoids can be accounted for the inhibition of NF-κB activation in brain ischemia-reperfusion insults [74,78,79,204,205], and in NPA-induced brain degeneration [48,139,140,141]. Since it has been shown that the IκB kinase can be directly activated by hydrogen peroxide and other ROS, the cellular antioxidant properties of flavonoids have been proposed to account for their inhibition of NF-κB activation [206,207,208]. Several works have shown that, as a result of the down-regulation of the NF-κB signaling pathway, kaempferol or its glycosides administration produces reduction of the infarct volume, of the expression of proinflammatory cytokines, of intercellular adhesion molecule-1, and inhibition of the activity of the enzymes iNOS, lipoxygenases, cyclooxygenase, and phospholipase A_2_ in MCAO ischemic models [78,79].

Therefore, the inhibition of microglial activation by kaempferol can, at least in part, account for its protective effects against ischemia-reperfusion and NPA-induced neurological dysfunctions associated with brain degeneration. Nevertheless, it should be noted that reactive astrocyte generation can also play a major role in the brain degeneration elicited by these insults. Indeed, Stanek et al. [209] have suggested that astrocyte dysfunction plays a critical role in HD pathogenesis on the basis of their results with the YAC128 mouse model. In addition, Wang et al. [210] have shown that reactive astrocytes can also further potentiate the activation of microglia in neurotoxins poisoning. Recently, we have demonstrated that the activation of NF-κB is the major molecular mechanism underlying the enhanced production of pro-inflammatory cytokines, interleukin-1α (IL-1α) and tumor necrosis factor α (TNFα), and complement component 1q (C1q) in NPA-induced degeneration of the *striatum* and the *hippocampus* [48]. These three cytokines, which are secreted by activated microglia, are necessary and sufficient to induce the generation of the highly neurotoxic reactive A1 astrocytes [147]. Kaempferol affords a strong protection against this feed-forward harmful cycle, since kaempferol blocked reactive A1 astrocytes generation in *striatum* and in the *hippocampus* of the rat model of NPA-induced brain degeneration [48]. This protective effect of kaempferol is of relevance for brain degeneration, because astrocytes, the most abundant brain cells, are necessary for neuronal survival and functioning, and, not less importantly, for the maintenance of the blood–brain barrier integrity [211].

The astrocytes can secrete pro-inflammatory mediators, inducing neuroinflammation with eventual disruption in tight junctions, which finally leads to blood–brain barrier integrity breakdown and brain edema [212,213]. Several works have shown that kaempferol actions helps to protect the blood–brain barrier integrity. It has been shown [78], in a rat model of MCAO ischemic stroke, that intragastrical administration of kaempferol inhibited monocyte chemoattractant protein-1 and intercellular adhesion molecule-1, proteins that potentiate the infiltration of immune system cells through the blood–brain barrier, and decreased matrix metalloproteinase-3 expression, a positive effect for preservation of blood–brain barrier integrity. Previously, Yu et al. [74] reported that IV administration of the kaempferol glycosides, kaempferol-3-O-rhamnoside and kaempferol-3-O-glucoside affords partial protection against blood–brain barrier neurovascular dysfunction in another rat model of MCAO ischemic stroke.

Finally, Zhang et al. [76] have recently proposed that post-stroke neuroinflammation is not only caused by microglia and astrocytes, but also by blood-derived white blood cells. These investigators focused their work on neutrophils, because infiltrating neutrophils produce pro-inflammatory cytokines, matrix metalloproteinases, nitric oxide, ROS and other cytotoxic molecules that accelerate brain damage [214]. The infiltration of neutrophils from the blood circulation into the brain is potentiated by the disruption of the blood–brain barrier by matrix metalloproteinases, aggravating brain injury and leading to high morbidity and mortality of cerebral ischemia-reperfusion [215]. Zhang et al. [76] have experimentally shown that kaempferol inhibits neutrophils activation, aggregation and infiltration into the brain, and preserved blood–brain barrier integrity in a cerebral ischemia-reperfusion rat MCAO model.

## 5. Conclusions

**Figure 6:**
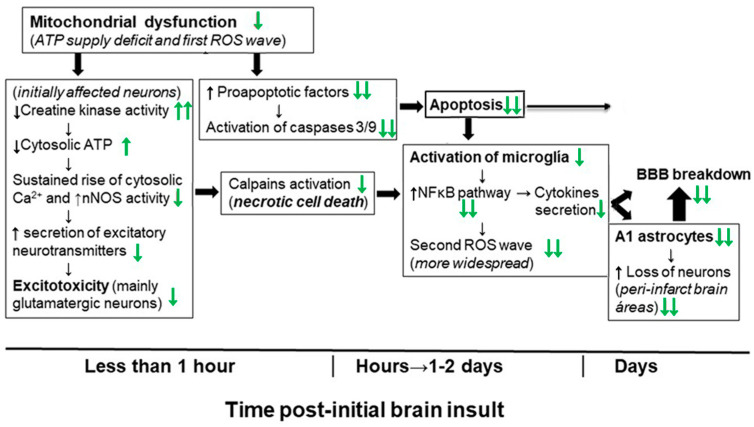
Schematic time course presentation of the major molecular and cellular mechanisms involved in brain damage induced by an ischemic brain stroke or by NPA injections, which has been shown to be strongly inhibited by kaempferol administration (indicated by green arrows) in experimental animal models, analyzed in detail in Section 4 of this review. Black arrows indicate the effects of ischemia-reperfusion and NPA administration. Two green arrows mean nearly complete inhibition by administration of kaempferol doses producing an efficient protection against brain damage and associated neurological dysfunctions. The time range of the initial events leading to caspases activation and necrotic neuronal death has been extrapolated to the brain from the data obtained using a variety of neuronal and cellular cultures in vitro. BBB means blood–brain barrier.

The accumulated experimental evidence with animal models allows us to conclude that kaempferol administration can efficiently protect against the neurological dysfunctions and brain damage induced by an ischemic brain stroke and by the neurotoxin NPA. Moreover, the main molecular and cellular mechanisms underlying the protective effects of kaempferol against neuronal and other brain cells death, and against brain degeneration induced by these insults seem to have been solidly established. These mechanisms are summarized in Figure 6. Notably, we noticed that both kaempferol and quercetin, a flavonol with a chemical structure only slightly different to that of kaempferol, inhibit the ATP hydrolytic activity of the rat brain mitochondrial F_1_,F_0_-ATPase without significant inhibition of the rate of oxygen consumption by mitochondria [45]. Remarkably, quercetin has been shown to act as an inhibitor of the ATP hydrolytic activity but not of the ATP synthesis activity of the mitochondrial F_1_,F_0_-ATPase, and it has been proposed that preventing the destruction of ATP by the mitochondrial F_1_,F_0_-ATPase affords cardioprotective benefit during cardiac ischemia [216]. Since quercetin also affords protection against ischemia-reperfusion-induced brain damage [1], the potential relevance of partial inhibition of the ATP hydrolytic activity of brain mitochondrial F_1_,F_0_-ATPase for the overall beneficial effect of kaempferol deserves to be further studied. Notably, kaempferol protects against the more rapid as well as the slow developing cell death mechanisms that have been shown to be responsible of the brain damage induced by an ischemic brain stroke and by the neurotoxin NPA. This points out that kaempferol administration can be beneficial not only in preventive treatments, but also in post-insult therapeutic treatments within the temporal windows of the slow developing stages of brain degeneration (apoptotic cell death, microglia activation and neurotoxic A1 astrocytes generation), which is more relevant for clinical applications. It should be noted that the delayed temporal windows lasts for several days after a transient brain ischemia stroke [51], as well as in experimental model animals treated with NPA [47].

## 6. Prospects

Taking into account the low toxicity of kaempferol for humans [201], the results obtained with ischemic MCAO stroke and with the neurotoxin NPA in experimental animal models strongly suggest the use of this flavonoid to improve the actual therapeutic treatments against ischemic brain stroke and Huntington’s disease. Of course, the sooner the kaempferol administration post-insult, the better it is in preventing brain damage leading to neurological dysfunctions, as this is expected to allow for a more efficient inhibition by kaempferol of the mechanisms leading to brain cells death and neuroinflammation. In this context, it is relevant to note here that kaempferol has been reported to stimulate the autophagy mechanism in cortical mice brain neurons with mitochondrial dysfunction [77], and to inhibit vascular endothelium inflammation [201], preventing irreversible vascular damage and hemorrhagic episodes in highly vascularized organs like the brain. In addition, the administration route of kaempferol is of particular relevance to define the dose of this flavonoid that needs to be administered for efficient protection against brain damage. Our results with experimental animal models indicate that to reach a similar extent of protection against brain damage, the doses of kaempferol needed are around 100-fold higher by intraperitoneal administration than by IV administration, a result that is consistent with the known poor oral bioavailability of kaempferol [201,217]. Also, IV administration allows for a more rapid and better accessibility of kaempferol to the brain, since it minimizes the extent of kaempferol metabolic processing before reaching the brain.

Brain inflammation and oxidative stress are cellular processes that mediate most neurodegenerative diseases, neurotoxic insults and other neuropathies. Therefore, it is not surprising that the beneficial actions of kaempferol have been reported for other neurodegenerative diseases, reviewed in [201,202], neurotoxins [218,219,220], and other neuropathies [221,222]. Our recent finding of inhibition of the generation of reactive neurotoxic A1 astrocytes by kaempferol reported in [48] further widens the human neurodegenerative diseases that can be a target for the therapeutic use of this flavonoid, as abundant A1 astrocytes have been found in the brain regions more prone to degeneration in HD, in Alzheimer’s and Parkinson’s diseases, and in amyotrophic lateral sclerosis and multiple sclerosis [147]. Notably, Wu et al. [223] have shown that complement C3, a selective biomarker of A1 astrocytes [147,224], is activated in human Alzheimer’s disease brain and is required for neurodegeneration in mouse models of amyloidosis and tauopathy. It must be recalled that NPA treatment induces tau pathology in the tangle-mouse model and also in wild type-mice [225]. Recently, we have reported that amyloid β peptides increase in the *striatum* in the hippocampus of NPA-treated rats [48]. These results point out the involvement of accepted hallmarks of Alzheimer’s disease in the NPA-induced neurodegenerative process. As shown in Lopez-Sanchez et al. [48], the administration of kaempferol prevents the increase of amyloid β peptides induced by NPA treatment. Interestingly, the IP administration of kaempferol has been reported to afford cognitive improvement in a mouse model of sporadic Alzheimer’s disease [226].

## Abbreviations

C1q	complement component 1q
GSH	reduced glutathione
HD	Huntington’s disease
IP	intraperitoneal
IV	intravenous
MCAO	middle cerebral artery occlusion
NF-κB	nuclear factor kappa light-chain enhancer of activated B cells
NOS (eNOS, iNOS and nNOS)	nitric oxide synthase (endotelial, inducible and neuronal isoforms)
NPA	3-nitropropionic acid
ROS	reactive oxygen species
TTC	2,3,5-triphenyltetrazolium chloride
TUNEL	terminal deoxyribonucleotidyl transferase-mediated dUTP-fluorescein nick-end labeling
